# Combinations of Histone Modifications Mark Exon Inclusion Levels

**DOI:** 10.1371/journal.pone.0029911

**Published:** 2012-01-05

**Authors:** Stefan Enroth, Susanne Bornelöv, Claes Wadelius, Jan Komorowski

**Affiliations:** 1 The Linnaeus Centre for Bioinformatics, Department of Cell and Molecular Biology, Science for Life Laboratory, Biomedical Center, Uppsala University, Uppsala, Sweden; 2 Science for Life Laboratory, Department of Immunology, Genetics and Pathology, Rudbeck Laboratory, Uppsala University, Uppsala, Sweden; 3 Interdisciplinary Centre for Mathematical and Computational Modelling, University of Warsaw, Warszawa, Poland; National University of Ireland Galway, Ireland

## Abstract

Splicing is a complex process regulated by sequence at the classical splice sites and other motifs in exons and introns with an enhancing or silencing effect. In addition, specific histone modifications on nucleosomes positioned over the exons have been shown to correlate both positively and negatively with exon expression. Here, we trained a model of “IF … THEN …” rules to predict exon inclusion levels in a transcript from histone modification patterns. Furthermore, we showed that combinations of histone modifications, in particular those residing on nucleosomes preceding or succeeding the exon, are better predictors of exon inclusion levels than single modifications. The resulting model was evaluated with cross validation and had an average accuracy of 72% for 27% of the exons, which demonstrates that epigenetic signals substantially mark alternative splicing.

## Introduction

The human genome contains around 20 000 genes and currently around 140 000 transcripts coding for different protein isoforms are known [1]. The process of concatenating the exons into a complete transcript, splicing, involves elimination of introns and specific exons and is performed by the spliceosome; a massive complex containing hundreds of proteins [2]. The constitution and function of the spliceosome is not yet fully known. The vast majority of eukaryotic introns end and start with specific sequences, AG and GT and these acceptor- and donor-sites constitute an invariant part of a signal by which specific subunits of the spliceosome can recognize the intron-exon boundaries [3]. On the mRNA-level, there are also exonic and intronic splicing enhancers (ESEs and ISEs) and silencers (ESSs and ISSs) [4], [5] These are short (6–8 nucleotides) sequence motifs that can be bound by proteins that further guide the splicing process. Recently, it has been suggested that in a given cell type, sequence information alone is enough to distinguish constitutively spliced exons from alternatively spliced exons [5]. However, this sequence-based system for splicing is not sufficient since different protein isoforms are produced by different cell types [6], and so the cell needs to regulate the splicing through a system not locked into the sequence itself. These epigenetic mechanisms are not the sole answer [7], but several DNA-binding proteins and chromatin remodelers have been shown to be important, and recently, post translational modifications to the histone proteins have been shown to, at least partly, regulate exon inclusion/exclusion [8], [9], [10], [11], [12] in gene transcripts. Conceptually, splicing can be achieved in two ways, either post-transcriptional or co-transcriptional. The classical textbook model is post-transcriptional where the whole mRNA is first transcribed and then the introns and, possibly, some exons are removed. Recently, the co-transcriptional model has been proposed [13], [14], [15], [16], [17] where inclusion/exclusion of a specific exon into the mRNA is decided before the whole mRNA is transcribed. The co-transcriptional model puts the spliceosome close to the DNA during transcription and it thus has the possibility to read and recognize the histone code.

Recently, a number of studies [8], [9], [10], [12], [18], [19] have shown genome-wide correlation between specific nucleosome modifications over internal exons and the exons expression and specifically, Luco *et al* [11] demonstrated histone modification mediated splice site selection in a set of genes. Taken together, this suggests an epigenetic signalling platform that could both serve as recognition of splice sites and determine inclusion and exclusion of exons into transcripts. Current studies on epigenetic control of expression focus on finding the strongest relations between a single histone modification and the expression of the exon and the combinatorial aspects have not yet been comprehensively addressed [20]. Here we present a combinatorial rule-based model that better reflects a part of the complex biological machinery behind splicing. Following our recent study [8] on nucleosome positioning and histone modifications over internal exons, we have now created a data-driven model which predicts exon inclusion levels from a binary (present/absent) representation of modifications to nucleosomes preceding, on, or succeeding the individual exon.

## Results and Discussion

The rule-based model was created using the most comprehensive data set on histone methylations and acetylations [21], [22] available to date. In all, 38 histone modifications are available in CD4+ T-cells and these where used to build a decision system with the modifications as attributes and exon inclusion or exclusion based on exon expression [23] data as decision. For each histone modification we considered regions preceding and succeeding the exons as well as centred over the exon giving a total of 114 attributes (3*38). Each histone modification was discretized as present or absent over the three regions (Methods). The generated rules are in the form of “IF … THEN ..” and a typical rule will read as “IF H2BK5me1 preceding exon is absent AND H3K4me1 succeeding exon is present AND H3K36me3 succeeding exon is absent AND H4K29me1 preceding exon is present THEN exon is excluded”. This means that the model is in human readable form and thus immediately interpretable and could be used as starting point for detailed experimental investigation on the interplay between histone modifications in relation to alternative splicing.

**Figure 1:**
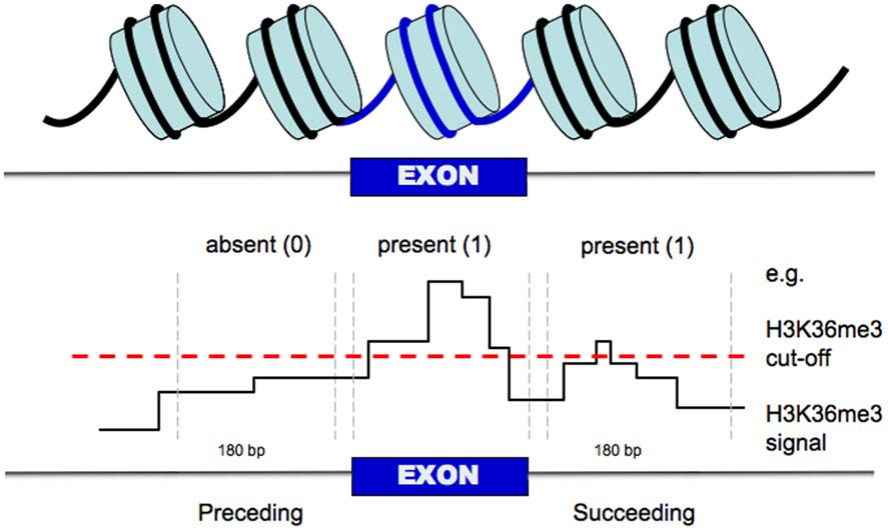
Schematic representation of the constructed data set. For each histone modification, e.g. H3K36me3, we deem it as present or absent in the region centred over the exon and the regions preceding and succeeding depending on if the histone modification pileup in such region is higher than a defined cut-off. This cut-off is specific for each histone modification since they were sequenced at difference depths.

The data set used to train the model was carefully constructed. The inclusion level of an exon in a gene was determined by first calculating the expression of the gene as an average over the ten highest expressed exons annotated to that gene. The exon inclusion level was then calculated as the quote between the exon expression and the average gene expression, and the exons with an inclusion level below 0.4 or between 0.9 and 1.1 were annotated as ‘spliced out’ or ‘included’, respectively. The exons were then filtered using several criteria. Firstly, we excluded the top 20% expressed genes as it has previously been postulated that, due to frequent polymerase II traffic, highly expressed genes are depleted of nucleosomes [24]. Secondly, we removed exons ranked first or last in any transcript and required that the exons were at least 50 bp long, flanked by at least 360 bp of intronic sequences (Methods). Lastly, we removed any exons that overlapped another exon. A schematic representation of the data is shown in Figure 1. The final data set of excluded exons contained 11 165 unique examples and we randomly selected the same number of unique exons from the 12 692 examples in the included class to get balanced classes for the training of the model. This was repeated ten times to catch variations of the results based on the selection of examples and the heuristic algorithms used for rule generation.

The attributes most contributing to the decisions were ranked using Monte Carlo feature selection [25] and the 20 highest ranked of the original 114 attributes were kept. Strikingly, modification to nucleosomes immediately preceding and succeeding the exon were often ranked higher than their counterparts ‘centred on’ exons and among the 20 selected attributes, only two were centred on the exon (Table S1). The 20 top-ranked attributes were used to build a rule-based classifier using the Rosetta system [26]. In order to prevent over-fitting of the rule model, we generated so-called approximate reducts and filtered the generated rules on both accuracy and support (Methods). The final model consisted of 165 rules and covered 27% of the selected exons. The performance of the primary model was assessed by a 10-fold cross validation schema for the ten data sets and yielded an average accuracy of 71.9% with a standard deviation of 0.3%. This accuracy is considerably higher than the 50% that would be expected by random guessing on the two equally sized data sets. Histone modifications previously [8], [9] identified as related to exon expression were present in the rules (e.g. H2BK5me1 and H4K20me1) as well as previously less well-studied modifications. However, the strongest univariate candidates [8], [9], [10], [11] e.g. H3K79me1, H3K79me3 and H3K36me3, were all selected as significant by the MCFS, but only H3K36me3 succeeding and preceding the exon were among the 20 highest ranked modifications and thus included in the rule model. Surprisingly, H3K36me3 was always required to be ‘absent’ in the rules for both decisions although it has previously been suggested that its presence is related to inclusion [8], [10], [11]. These results lead us to investigate the properties of the H3K36me3 histone modification in particular and we could conclude that the distribution of this specific mark was too similar (Table S2) over both our classes for its presence/absence to constitute a rule in itself. When H3K36me3 was explicitly absent, however, it the model was able to distinguish between ‘included’ and ‘spliced out’ exons using combinations with other marks.

**Figure 2:**
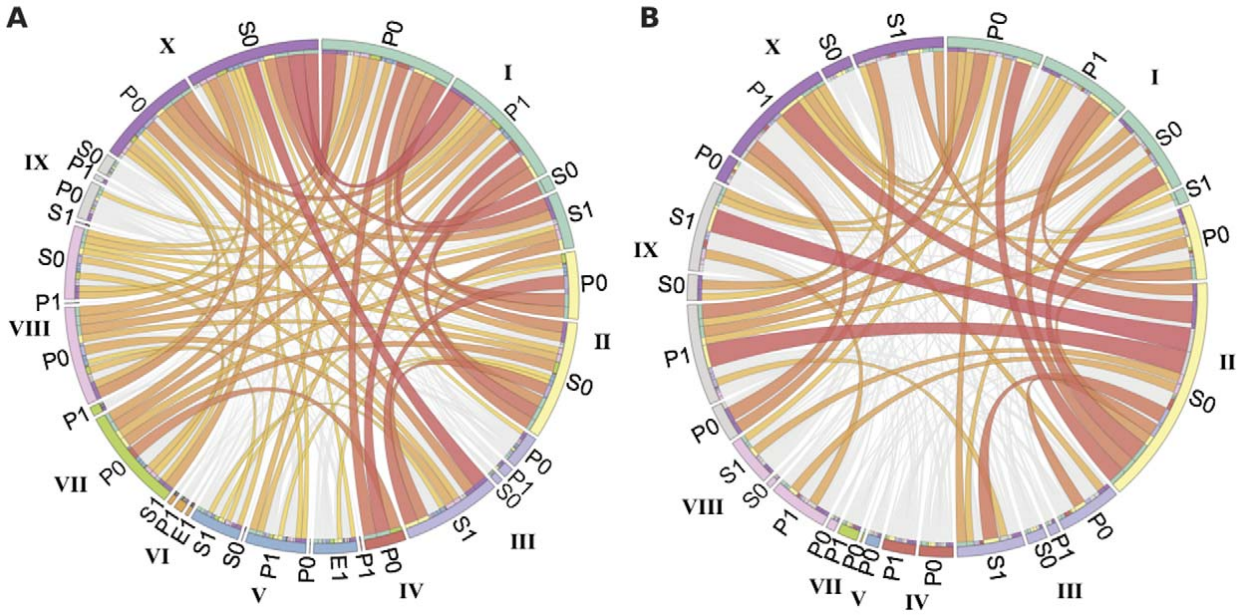
Pair-wise co-occurrence of histone modifications in the model. (**A**) Rules predicting ‘spliced out’. Any each histone modification that occurs in any rule is labelled on the outer ring, and co-occurrence with other histone modifications are illustrated by ribbons across the circle. The width of the ribbon correlates to the number of rules where the two modifications co-occur as attributes. Colouring of the ribbons indicates ranking of number of rules with the given pair over the whole model with the lower 75% coloured in grey. The colours of the inner ring correspond to the colour in the outer ring in the other end of a given ribbon. The figure labels are ‘I’: H2BK5me1, ‘II’: H3K36me3, ‘III’: H3K4me1, ‘IV’: H3K9me1, ‘V’: H3K9me2, ‘VI’: H3K9me3, ‘VII’: H3R2me1, ‘VIII’: H4K16ac, ‘IX’: H4K20me1, ‘X’: H4K91ac; S: succeeding the exon, P: preceding the exon, E: on the exon; 0: is absent, 1: is present (e.g. “A P0” is interpreted as “H2BK5me1 preceding the exon is absent”). (**B**) Same as (A) but for the rules predicting ‘included’ exons. Images was generated using Circos [29].

In the model, the number of condition attributes in the rules varied between two and nine (Figure S1). The most common number of condition attributes was five (56%) for the ‘spliced out’ class and six to eight (75%) for the ‘included’ class. In total 66% of the rules in contained 6–8 condition attributes and no rules consisted of a single attribute. Only 6 rules out of the total 165 did not require at least one histone modification to be absent from the region. The complexity of the interplay between histone modifications is illustrated in Figure 2A, demonstrating pair-wise co-occurrence of attributes in the rules predicting ‘spliced out’. We calculated the predicted accuracy of the rules assuming that the histone modifications were independent from each other (Methods). If the accuracy of a rule is greater than the predicted sum of its parts, this corresponds to rules where the combination indeed gives more information about the exon inclusion level than what can be obtained using only one attribute at a time. If it is not, it could be explained by correlation between attributes in the rules, or by rules that only summarize the contribution of independent attributes. Many of the histone modifications are indeed expected to be correlated and, moreover, modifications present e.g. centred on the exon are often accompanied by modifications in the flanking introns and vice versa.

**Figure 3:**
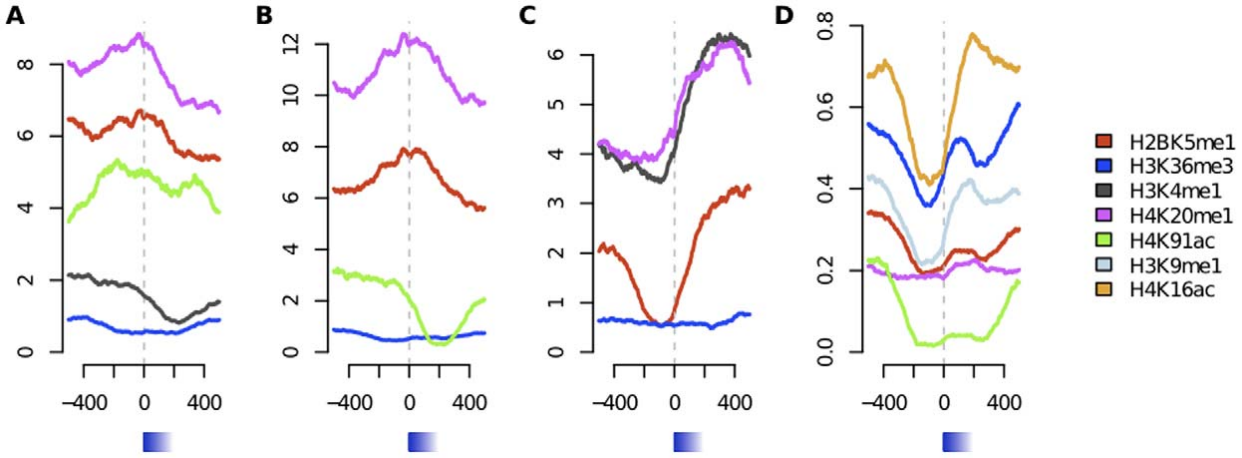
Footprints of rules from the model. The footprints are centred on intron-exon junctions (I/E) with examples of rules in the model from the (A) ‘Spliced out’ class (covers 358 exons, −6 pp accuracy gain over independent histone, referred to as S1 in Table 1), (B) ‘Spliced out’ (296, +5 pp, S3), (C) ‘Spliced out’ (260, +12 pp, S5) and (D) ‘Included’ (3738, −8 pp, I1). The number of reads normalized to sequence depth as left axis.

We observed an overall combinatorial effect for the ‘spliced out’ class where 23 out of the 32 rules had a combinatorial gain in accuracy (Table S3) and 4 of them had a gain of more than 10 percentage units. This suggests that the attributes, e.g. histone modifications, in rules predicting ‘spliced out’ exons share control of exon inclusion levels and are not only independent correlations taken together. The rules for the ‘included’ class were of a different character. They contained, in general, more attributes and many of these were required to be absent. Only 2 out of the 133 rules showed a combinatorial gain, and we did not find specific patterns for inclusion, but rather a description of the background state exons without any ‘spliced out’ signals. The pair wise co-occurrence of attributes for the ‘included’ class is illustrated in Figure 2B. The five top-ranked rules for both classes are shown in Table 1, and footprints for the exons that fulfilled some of these are shown in Figure 3A–D. Note that there all histone modification signals were low when the class is ‘included’ (Figure 3D), which confirmed that this correspond to a background state of an exon.

**Table 1:** Top-ranked rules.

ID	LHS Rule	Support	Accuracy	Comb gain	P-value
S1	H2BK5me1.prec = 1 H2BK5me1.succ = 1 H3K4me1.succ = 0 H3K36me3.prec = 0 H3K36me3.succ = 0 H4K20me1.prec = 1 H4K91ac.prec = 1	358	0.737	−6.1	1.54E-025
S2	H3K9me1.prec = 1 H3K4me1.prec = 0 H3K36me3.succ = 0 H4K20me1.succ = 1 H4K91ac.prec = 1	307	0.733	+1.4	2.18E-021
S3	H2BK5me1.prec = 1 H2BK5me1.succ = 1 H3K36me3.prec = 0 H3K36me3.succ = 0 H4K20me1.prec = 1 H4K91ac.succ = 0	296	0.736	+4.6	3.30E-021
S4	H3K4me1.prec = 0 H3K4me1.succ = 1 H3K36me3.succ = 0 H4K20me1.prec = 1 H4K91ac.prec = 1	195	0.785	+4.4	9.48E-020
S5	H2BK5me1.prec = 0 H3K4me1.succ = 1 H3K36me3.succ = 0 H4K20me1.succ = 1	260	0.738	+11.8	4.62E-019
I1	H2BK5me1.prec = 0 H2BK5me1.succ = 0 H3K9me1.prec = 0 H3K36me3.prec = 0 H3K36me3.succ = 0 H4K16ac.prec = 0 H4K20me1.prec = 0 H4K91ac.prec = 0 H4K91ac.succ = 0	3738	0.775	−7.5	6.99E-244
I2	H2BK5me1.prec = 0 H2BK5me1.succ = 0 H3K9me1.prec = 0 H3K36me3.prec = 0 H3K36me3.succ = 0 H4K16ac.prec = 0 H4K20me1.succ = 0 H4K91ac.prec = 0 H4K91ac.succ = 0	3737	0.774	−7.7	5.28E-242
I3	H2BK5me1.prec = 0 H2BK5me1.succ = 0 H3R2me1.prec = 0 H3K9me1.prec = 0 H3K4me1.succ = 0 H3K36me3.prec = 0 H3K36me3.succ = 0 H4K91ac.prec = 0 H4K91ac.succ = 0	3282	0.784	−5.6	4.74E-227
I4	H2BK5me1.prec = 0 H2BK5me1.succ = 0 H3R2me1.prec = 0 H3K9me1.prec = 0 H3K36me3.prec = 0 H3K36me3.succ = 0 H4K16ac.prec = 0 H4K20me1.prec = 0 H4K91ac.succ = 0	3026	0.783	−5.9	2.72E-205
I5	H2BK5me1.prec = 0 H2BK5me1.succ = 0 H3R2me1.prec = 0 H3K9me1.prec = 0 H3K36me3.prec = 0 H3K36me3.succ = 0 H4K16ac.prec = 0 H4K20me1.succ = 0 H4K91ac.succ = 0	3024	0.782	−6.0	1.22E-202

Top ranked rules in the model for ‘Spliced out’ (top) and ‘Included’ (bottom). The ID column contain a rule identifier in which the first letter indicate the rule outcome (S for ‘spliced out’ and I for ‘included’). The left-hand side of the rule (LHS Rule) is the conditions in the IF-part of the rule. The support is defined as the number of examples in the data that are covered by the IF-part of the rule and the accuracy is the proportion of the correct decision among those. The combinatorial gain (Comb gain) is the change in accuracy (percentage points) compared to the theoretical accuracy, assuming independent modifications. The P-value denotes the probability of the rule calculated from a hypergeometric distribution.

Due to the fact that the model was trained only on exons that did not overlap one another, we expect it to explain splicing of cassette type that can be estimated on cell population level from expression data. The model thus cannot consider e.g. splicing caused by alternative exon boundaries and such events needs to be formulated as a classification problem and additional investigations are required to assess whether other splicing patterns can be predicted using out approach.

In conclusion, we have shown that a substantial proportion of alternative splicing events can be attributed to the combinatorial status of histone modifications on nucleosomes preceding, on, or succeeding the exon and that combination of specific histone modifications are often better predictors of exon inclusion levels than single histone modifications.

## Methods

### Experimental Data

The data used here was based on ChIP-seq histone modification data for 38 methylations and acetylations [21], [22] in CD4+ T cell together with exon expression data [23].

A signal assembly for the ChIP-seq fragments was done as described by Andersson *et al.* [8] and individual fragments were extended to 150 bp and pileup signals was stored in a binary format [27]. To discretize the modification signals to binary (present/absent) attributes for three positions in relation to the exon (preceding, on or succeeding the exon), exons and the closest 180 bp of flanking intronic regions were searched for significant enrichment of the histone modification signals, assuming a Poisson distribution (p<0.05) of the fragments. 80% of the genome was considered mappable in the calculation of the mean signal for the Poisson distribution. The 20 bp closest to the intron/exon or exon/intron junction on both sides were excluded from the search.

We considered only internal exons, that is we excluded all exons that where annotated as first or last in any transcript. Exons longer than 50 bp with flanking introns longer than 360 bp and no overlap to other exons were identified. As introns of more than 360 bps were required in the exon selection, the intronic regions selected for consecutive exon could never overlap one another. The gene expression may theoretically be approximated as the highest expression of an exon in the gene, but to avoid high noise impact the gene expression was calculated from the exon expression as the average of the ten highest expressed exons in the gene. The 20% highest expressed genes were excluded from the study and the inclusion levels of the remaining exons were calculated as the exon expression divided by the gene expression. Genes with only one exon were excluded and among the remaining exons 13 374 which had an inclusion level below 0.4 were annotated as ‘spliced out’ and 11 587 which had an inclusion level between 0.9 and 1.1 were annotated as ‘included’. Exons with no modifications present were removed from the data, which gave in total 12 692 ‘included’ and 11 165 ‘spliced out’ exons. All annotation data on exons were taken from the Ensembl [28] system (*H.Sapiens* 54_36p).

### Model generation and validation

The decision table was created with exons from all autosomes, taking the histone modifications as condition attributes and the exon inclusion level as decision. The two inclusion level classes ‘spliced out’ and ‘included’ were roughly of similar size why all those exons were used in the Monte Carlo feature selection (MCFS). The MCFS was performed using dmLab 1.70 [25] with the parameters *s* = 5 000, *m* = 10, *t* = 5, *u* = 0 and *v* = 1. The remaining settings were mcfs.classifier = j48, mcfs.splitRatio = 0.66, mcfs.balanceClasses = true, mcfs.balanceRartio = 1, mcfs.cutPointRuns = 30, mcfs.cutPointAlpha = 0.05 and j48.useInfoGain = false. The rankings are shown in Table S1. Out of 114 attributes, 94 were found significant (p<0.05) using a randomization test. The 20 highest ranked attributes were kept for the rule generation step.

Rules were generated in Rosetta [26] with the JohnsonReducer, relative to the objects and using approximate reducts with a hitting fraction of 0.80. All 11 165 exons from the ‘spliced out’ class were selected together with approximately the same number from the ‘included’ class to define a training set with an equal number of examples from each decision class. This was repeated ten times to construct ten different data sets. On each of those data sets, a rule model was trained and a 10-fold cross validation was performed to assess the performance of the rule model. The cross validation results were averaged and the rules trained on all data sets were merged together, and rule accuracy and support were re-calculated on the original data. The support was calculated as the number of examples that fit the conditions on the left-hand side of the rule, and accuracy as the number of correctly classified objects by the rule divided by the rule support.

Footprints visualizing the mean modification signal-per-bp for all exons supporting each rule were created using the SICTIN software [27]. The number of exons that is covered by a rule was defined as the number of exons that fulfil the IF-part of the rule.

The predicted rule accuracy was calculated assuming linear independence between the attributes and was calculated as |*T*|*p^T^*_1_ *p^T^*_2_…*p^T^_n_*/(|*T*|*p^T^*_1_ *p^T^*_2_…*p^T^_n_*+|*F*|*p^F^*_1_ *p^F^*_2_…*p^F^_n_*) were *T* is the set of objects in the decision class of the rule, *F* is the objects in the other classes, and *p^C^_i_* is the probability that an object from class *C* fulfil condition number *i*. The probabilities were estimated by counting the proportion of objects from each class that passed each condition.

## Supporting Information

Figure S1 **Rule length.** The number of rules for the classes ‘Spliced out’ and ‘Included’ shown split on the number of attributes in the LHS of the rules.
(TIF)

Table S1 **Ranking of the histone modifications by their relative importance (RI).** The P-value shows the significance of the RIs relative the permutation test. The 20 highest ranked attributes (marked by an ‘x’ to the left) were kept for rule generation.
(DOC)

Table S2 **Exon inclusion depending on the presence of histone modifications.** Inclusion percent is defined as the percentage of exons from the ‘included’ class.
(DOC)

Table S3 **All rules in the classifier.** Each rule is represented as a list of the conditions in the rule (Rule) and the decision outcome (Class). The support is defined as the number of examples in the data that are covered by the IF-part of the rule and the accuracy is the proportion of the correct decision among those. The theoretical accuracy was calculated assuming independent modifications, and the combinatorial gain (Comb gain) is the change in accuracy (percentage points) between the accuracy and the theoretical accuracy. The P-value denotes the probability of the rule calculated from a hypergeometric distribution.
(XLS)
