# Supplementary figures and images for: Combinations of Histone Modifications Mark Exon Inclusion Levels
Source: PLoS One. 2012 Jan 5;7(1):e29911. doi: 10.1371/journal.pone.0029911 (PMC3252363; doi:10.1371/journal.pone.0029911)

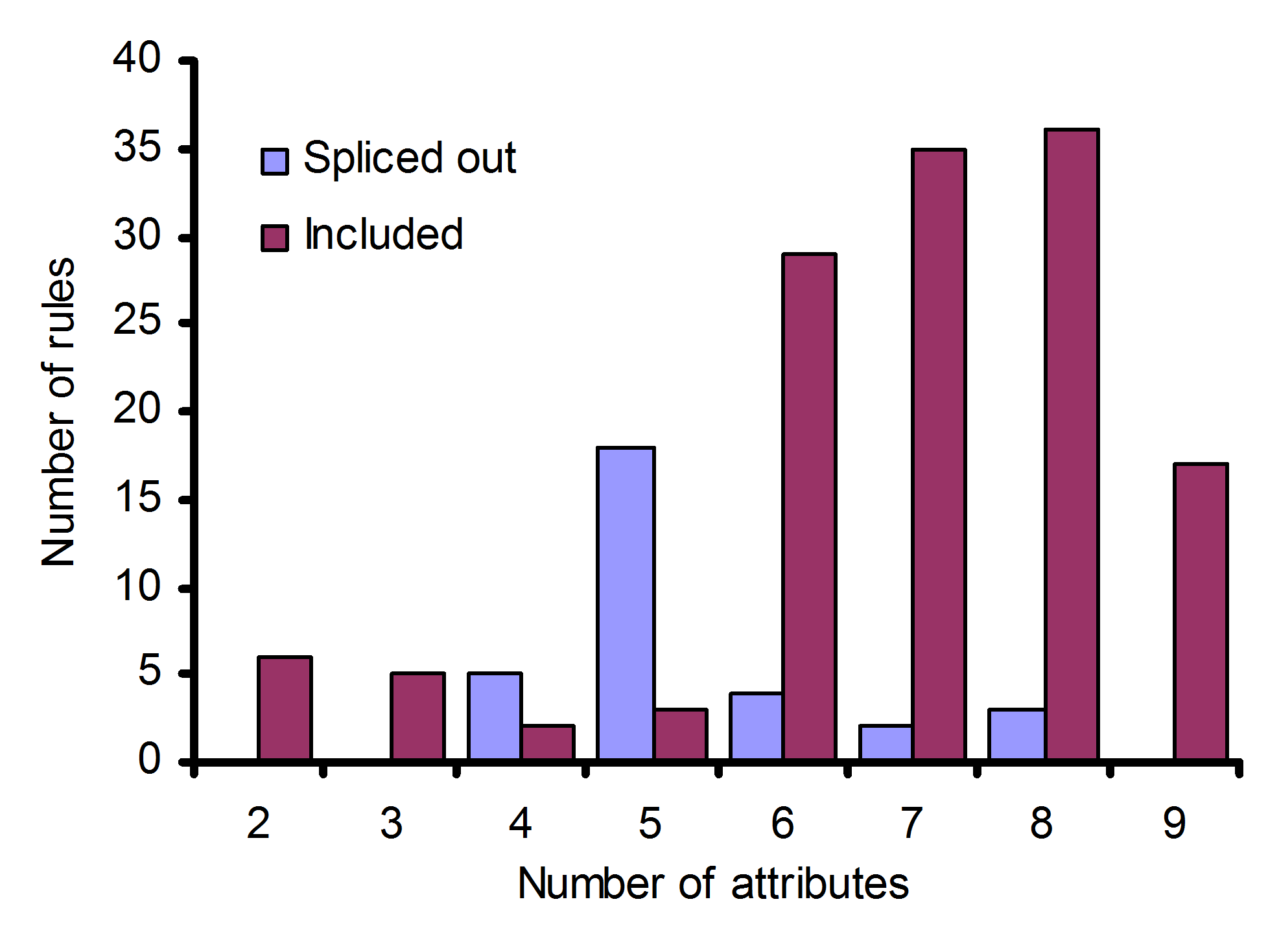

Supplement: Figure S1 — Rule length. The number of rules for the classes ‘Spliced out’ and ‘Included’ shown split on the number of attributes in the LHS of the rules. (TIF) [file pone.0029911.s001.tif]
