# Supplementary material for: Combinations of Histone Modifications Mark Exon Inclusion Levels
Source: PLoS One. 2012 Jan 5;7(1):e29911. doi: 10.1371/journal.pone.0029911 (PMC3252363; doi:10.1371/journal.pone.0029911)
Supplement: Table S1 — Ranking of the histone modifications by their relative importance (RI). The P-value shows the significance of the RIs relative the permutation test. The 20 highest ranked attributes (marked by an ‘x’ to the left) were kept for rule generation. (DOC) [file pone.0029911.s002.doc]

|  | **Histone modification** | **RI** | **RI permutation mean (stdev)** | **P-value** |
| --- | --- | --- | --- | --- |
| x | H3K9me2 | 0.0170 | 0.0014(0.0001) | 0.00E+00 |
| x | H4K91ac.succ | 0.0168 | 0.0023(0.0001) | 0.00E+00 |
| x | H4K91ac.prec | 0.0157 | 0.0023(0.0001) | 0.00E+00 |
| x | H2BK5me1.succ | 0.0155 | 0.0023(0.0001) | 0.00E+00 |
| x | H2BK5me1.prec | 0.0142 | 0.0023(0.0001) | 0.00E+00 |
| x | H4K20me1.succ | 0.0129 | 0.0019(0.0001) | 0.00E+00 |
| x | H4K20me1.prec | 0.0125 | 0.0019(0.0001) | 0.00E+00 |
| x | H3K9me2.prec | 0.0117 | 0.0016(0.0001) | 0.00E+00 |
| x | H3K9me2.succ | 0.0100 | 0.0017(0.0001) | 0.00E+00 |
| x | H4K16ac.prec | 0.0086 | 0.0025(0.0002) | 0.00E+00 |
| x | H4K16ac.succ | 0.0076 | 0.0024(0.0002) | 0.00E+00 |
| x | H3K9me3.prec | 0.0074 | 0.0008(0) | 0.00E+00 |
| x | H3K9me3.succ | 0.0070 | 0.0008(0.0001) | 0.00E+00 |
| x | H3K36me3.succ | 0.0062 | 0.0025(0.0002) | 0.00E+00 |
| x | H3K36me3.prec | 0.0060 | 0.0024(0.0001) | 0.00E+00 |
| x | H3K9me3 | 0.0058 | 0.0006(0) | 0.00E+00 |
| x | H3K4me1.prec | 0.0056 | 0.0018(0.0001) | 0.00E+00 |
| x | H3K9me1.prec | 0.0055 | 0.0024(0.0001) | 0.00E+00 |
| x | H3R2me1.prec | 0.0054 | 0.0026(0.0001) | 0.00E+00 |
| x | H3K4me1.succ | 0.0053 | 0.0019(0.0001) | 0.00E+00 |
|  | H3K23ac | 0.0051 | 0.0019(0.0001) | 0.00E+00 |
|  | H3K9me1.succ | 0.0049 | 0.0024(0.0001) | 0.00E+00 |
|  | H3K27me3 | 0.0048 | 0.0017(0.0001) | 0.00E+00 |
|  | H2BK20ac.prec | 0.0046 | 0.0025(0.0001) | 0.00E+00 |
|  | H3K27me2 | 0.0043 | 0.0019(0.0001) | 0.00E+00 |
|  | H3K79me1.succ | 0.0041 | 0.002(0.0001) | 0.00E+00 |
|  | H4K91ac | 0.0040 | 0.002(0.0001) | 0.00E+00 |
|  | H2BK20ac.succ | 0.0039 | 0.0025(0.0001) | 0.00E+00 |
|  | H2BK120ac.succ | 0.0039 | 0.0023(0.0001) | 0.00E+00 |
|  | H2BK5me1 | 0.0039 | 0.0021(0.0001) | 0.00E+00 |
|  | H3K18ac.prec | 0.0038 | 0.0024(0.0001) | 0.00E+00 |
|  | H3K27me1.prec | 0.0038 | 0.0022(0.0001) | 0.00E+00 |
|  | H3R2me1.succ | 0.0038 | 0.0026(0.0002) | 1.00E-10 |
|  | H3K18ac.succ | 0.0038 | 0.0024(0.0001) | 0.00E+00 |
|  | H4K20me1 | 0.0037 | 0.0014(0.0001) | 0.00E+00 |
|  | H3K4ac.prec | 0.0037 | 0.0025(0.0001) | 0.00E+00 |
|  | H3K27me1.succ | 0.0037 | 0.0022(0.0001) | 0.00E+00 |
|  | H2BK120ac.prec | 0.0036 | 0.0023(0.0001) | 0.00E+00 |
|  | H3K36me3 | 0.0036 | 0.0022(0.0001) | 0.00E+00 |
|  | H3K23ac.prec | 0.0035 | 0.0023(0.0001) | 0.00E+00 |
|  | H3K14ac | 0.0034 | 0.0021(0.0002) | 0.00E+00 |
|  | H3K4me2.succ | 0.0034 | 0.0018(0.0001) | 0.00E+00 |
|  | H3K79me1.prec | 0.0033 | 0.0019(0.0001) | 0.00E+00 |
|  | H3K4ac.succ | 0.0033 | 0.0025(0.0001) | 2.10E-09 |
|  | H3K27me1 | 0.0033 | 0.0017(0.0001) | 0.00E+00 |
|  | H3K4me2.prec | 0.0033 | 0.0019(0.0001) | 0.00E+00 |
|  | H3K23ac.succ | 0.0032 | 0.0024(0.0001) | 0.00E+00 |
|  | H3K27me3.succ | 0.0031 | 0.002(0.0001) | 0.00E+00 |
|  | H3K36me1 | 0.0030 | 0.002(0.0001) | 0.00E+00 |
|  | H2AK5ac.prec | 0.0030 | 0.0026(0.0001) | 2.13E-06 |
|  | H2AK5ac.succ | 0.0029 | 0.0025(0.0001) | 2.57E-03 |
|  | H4K16ac | 0.0028 | 0.0021(0.0001) | 0.00E+00 |
|  | H4K5ac.succ | 0.0028 | 0.0025(0.0001) | 1.60E-02 |
|  | H4K5ac.prec | 0.0028 | 0.0025(0.0001) | 1.21E-02 |
|  | H4K8ac.succ | 0.0027 | 0.0026(0.0001) | 1.62E-01 |
|  | H3K9ac | 0.0027 | 0.0021(0.0001) | 4.23E-08 |
|  | H4K5ac | 0.0026 | 0.0021(0.0002) | 4.05E-03 |
|  | H4K8ac.prec | 0.0026 | 0.0026(0.0001) | 2.52E-01 |
|  | H2AK5ac | 0.0026 | 0.0022(0.0001) | 2.54E-04 |
|  | H4K8ac | 0.0026 | 0.0022(0.0001) | 2.96E-03 |
|  | H3K14ac.prec | 0.0026 | 0.0026(0.0001) | 3.48E-01 |
|  | H3K9me1 | 0.0026 | 0.002(0.0001) | 1.20E-09 |
|  | H4K12ac.succ | 0.0026 | 0.0026(0.0001) | 4.70E-01 |
|  | H3K27me3.prec | 0.0026 | 0.002(0.0001) | 5.08E-07 |
|  | H3R2me1 | 0.0026 | 0.0023(0.0001) | 1.43E-03 |
|  | H4K12ac.prec | 0.0025 | 0.0026(0.0001) | 6.15E-01 |
|  | H4R3me2 | 0.0025 | 0.0017(0.0001) | 1.00E-10 |
|  | H3K36me1.succ | 0.0025 | 0.0024(0.0001) | 1.84E-01 |
|  | H3K36me1.prec | 0.0025 | 0.0024(0.0001) | 6.00E-02 |
|  | H3K14ac.succ | 0.0025 | 0.0025(0.0002) | 4.63E-01 |
|  | H3K9ac.prec | 0.0025 | 0.0025(0.0002) | 5.06E-01 |
|  | H2BK12ac.prec | 0.0025 | 0.0025(0.0002) | 4.18E-01 |
|  | H3K9ac.succ | 0.0025 | 0.0026(0.0001) | 7.64E-01 |
|  | H2BK5ac.prec | 0.0025 | 0.0022(0.0002) | 4.85E-02 |
|  | H3K27ac.prec | 0.0025 | 0.0022(0.0001) | 1.69E-02 |
|  | H3K27me2.prec | 0.0025 | 0.0022(0.0001) | 1.04E-02 |
|  | H2BK12ac.succ | 0.0025 | 0.0025(0.0001) | 5.19E-01 |
|  | H2BK5ac.succ | 0.0024 | 0.0022(0.0001) | 3.92E-02 |
|  | H3K27ac.succ | 0.0024 | 0.0022(0.0002) | 1.15E-01 |
|  | H2BK20ac | 0.0024 | 0.0021(0.0002) | 2.05E-02 |
|  | H3K79me1 | 0.0024 | 0.0015(0.0001) | 0.00E+00 |
|  | H4K12ac | 0.0024 | 0.0022(0.0001) | 5.05E-02 |
|  | H2BK12ac | 0.0024 | 0.002(0.0001) | 1.67E-04 |
|  | H3K27me2.succ | 0.0024 | 0.0022(0.0001) | 1.13E-01 |
|  | H4R3me2.succ | 0.0024 | 0.0022(0.0002) | 1.30E-01 |
|  | H3R2me2.prec | 0.0024 | 0.0022(0.0001) | 5.95E-03 |
|  | H4R3me2.prec | 0.0023 | 0.0022(0.0002) | 2.45E-01 |
|  | H3R2me2.succ | 0.0023 | 0.0022(0.0001) | 7.09E-02 |
|  | H3K4ac | 0.0023 | 0.0021(0.0001) | 2.22E-02 |
|  | H2AK9ac.succ | 0.0023 | 0.0022(0.0001) | 2.23E-01 |
|  | H3K18ac | 0.0023 | 0.002(0.0001) | 1.54E-02 |
|  | H2BK120ac | 0.0022 | 0.0019(0.0001) | 5.22E-05 |
|  | H3K4me1 | 0.0022 | 0.0015(0.0001) | 0.00E+00 |
|  | H2AK9ac.prec | 0.0022 | 0.0022(0.0001) | 3.58E-01 |
|  | H2BK5ac | 0.0021 | 0.0017(0.0001) | 1.22E-03 |
|  | H3K27ac | 0.0021 | 0.0017(0.0001) | 2.17E-03 |
|  | H3K4me2 | 0.0021 | 0.0014(0.0001) | 0.00E+00 |
|  | H3K4me3.succ | 0.0020 | 0.0011(0.0001) | 0.00E+00 |
|  | H3K79me3.succ | 0.0020 | 0.0015(0.0001) | 1.87E-06 |
|  | H3K79me3.prec | 0.0019 | 0.0014(0.0001) | 2.00E-10 |
|  | H2AK9ac | 0.0019 | 0.0017(0.0001) | 9.91E-03 |
|  | H3R2me2 | 0.0019 | 0.0016(0.0001) | 3.40E-03 |
|  | H3K4me3.prec | 0.0019 | 0.0011(0.0001) | 0.00E+00 |
|  | H3K36ac.prec | 0.0019 | 0.0015(0.0001) | 1.02E-04 |
|  | H3K36ac.succ | 0.0018 | 0.0015(0.0001) | 2.05E-04 |
|  | H3K79me2.prec | 0.0017 | 0.001(0) | 0.00E+00 |
|  | H3K79me2.succ | 0.0017 | 0.001(0.0001) | 0.00E+00 |
|  | H3K36ac | 0.0016 | 0.001(0.0001) | 1.50E-09 |
|  | H3K79me3 | 0.0016 | 0.001(0.0001) | 1.25E-08 |
|  | H4K20me3.prec | 0.0013 | 0.0009(0.0001) | 0.00E+00 |
|  | H3K4me3 | 0.0013 | 0.0008(0) | 0.00E+00 |
|  | H4K20me3.succ | 0.0013 | 0.0009(0.0001) | 2.00E-10 |
|  | H3K79me2 | 0.0012 | 0.0007(0) | 0.00E+00 |
|  | H4K20me3 | 0.0011 | 0.0007(0) | 0.00E+00 |
