# Supplementary material for: Combinations of Histone Modifications Mark Exon Inclusion Levels
Source: PLoS One. 2012 Jan 5;7(1):e29911. doi: 10.1371/journal.pone.0029911 (PMC3252363; doi:10.1371/journal.pone.0029911)
Supplement: Table S2 — Exon inclusion depending on the presence of histone modifications. Inclusion percent is defined as the percentage of exons from the ‘included’ class. (DOC) [file pone.0029911.s003.doc]

| **Modification** | **Inclusion (%) with modification** | **Inclusion (%) without modification** |
| --- | --- | --- |
| H3K36me3.prec | 50 | 55 |
| H3K36me3 | 54 | 53 |
| H3K36me3.succ | 51 | 55 |
